# MiR‐203 improves cardiac dysfunction by targeting PARP1‐NAD ^+^ axis in aging murine

**DOI:** 10.1111/acel.14063

**Published:** 2023-12-14

**Authors:** Limin Zhao, Pingping Tang, Yuan Lin, Menghan Du, Huimin Li, Lintong Jiang, Henghui Xu, Heyang Sun, Jingjing Han, Zeqi Sun, Run Xu, Han Lou, Zhouxiu Chen, Philipp Kopylov, Xin Liu, Yong Zhang

**Affiliations:** ^1^ Department of Pharmacology (State‐Province Key Laboratories of Biomedicine‐ Pharmaceutics of China, Key Laboratory of Cardiovascular Research, Ministry of Education), College of Pharmacy Harbin Medical University Harbin China; ^2^ Department of Pharmacy The Fourth Affiliated Hospital of Harbin Medical University Harbin China; ^3^ Department of Pharmacy Caoxian People's Hospital Heze China; ^4^ Department of Preventive and Emergency Cardiology Sechenov First Moscow State Medical University Moscow Russian Federation; ^5^ National Key Laboratory of Frigid Zone Cardiovascular Diseases (NKLFZCD) Harbin China; ^6^ Research Unit of Noninfectious Chronic Diseases in Frigid Zone Chinese Academy of Medical Sciences Harbin China; ^7^ Institute of Metabolic Disease Heilongjiang Academy of Medical Science Harbin China

**Keywords:** heart aging, miR‐203, mitochondrial function, NAD^+^, PARP1

## Abstract

Heart aging is a prevalent cause of cardiovascular diseases among the elderly. NAD^+^ depletion is a hallmark feature of aging heart, however, the molecular mechanisms that affect NAD^+^ depletion remain unclear. In this study, we identified microRNA‐203 (miR‐203) as a senescence‐associated microRNA that regulates NAD^+^ homeostasis. We found that the blood miR‐203 level negatively correlated with human age and its expression significantly decreased in the hearts of aged mice and senescent cardiomyocytes. Transgenic mice with overexpressed miR‐203 (TgN (miR‐203)) showed resistance to aging‐induced cardiac diastolic dysfunction, cardiac remodeling, and myocardial senescence. At the cellular level, overexpression of miR‐203 significantly prevented D‐gal‐induced cardiomyocyte senescence and mitochondrial damage, while miR‐203 knockdown aggravated these effects. Mechanistically, miR‐203 inhibited PARP1 expression by targeting its 3′UTR, which helped to reduce NAD^+^ depletion and improve mitochondrial function and cell senescence. Overall, our study first identified miR‐203 as a genetic tool for anti‐heart aging by restoring NAD^+^ function in cardiomyocytes.

## Graphical abstract

With advancing age, the expression of miR‐203 markedly decreases. MiR‐203 downregulates PARP1 expression by specifically targeting its 3′UTR, leading to a mitigation of NAD^+^ depletion and enhancement of mitochondrial function, ultimately mitigating cellular senescence. Our research highlights miR‐203 as a genetic tool for improving cardiac dysfunction by reinstating NAD^+^ levels in aging murine.

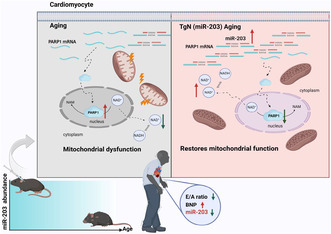

**Abbreviations**
COPD: chronic obstructive pulmonary disease
CVDs: cardiovascular diseases
DBP: diastolic blood pressure
DCM: diabetic cardiomyopathy
D‐gal: D‐galactose
E/A: early‐to‐late transmitral filling velocity ratio
FISH: fluorescence in situ hybridization
HFpEF: heart failure with preserved ejection fraction
HW/BW: heart weight/body weight
I/R: ischemia–reperfusion
IVRT: isovolumic relaxation time
LVDD: left ventricular diastolic dysfunction
LVEF: left ventricular ejection fraction
LVPW: left ventricular posterior wall
MI: myocardial infarction
miR‐203: microRNA‐203
mRNA: messenger RNA
NAD^+^
: nicotinamide adenine dinucleotide
NAM: NAD^+^ precursor nicotinamide
NMCM: neonatal mouse cardiomyocyte
NMN: nicotinamide mononucleotide
PARP1: Poly (ADP‐ribose) polymerase 1
ROS: reactive oxygen species
SA‐β‐Gal: senescence‐associated β‐galactosidase
SBP: systolic blood pressure
TEM: transmission electron microscopy
UTR: untranslated region
WT: wild type

## 1 INTRODUCTION

Aging is a major risk factor for cardiovascular diseases (CVDs), which is a leading cause of morbidity and mortality worldwide (Marian et al., 2018; Triposkiadis et al., 2019). Epidemiological studies have shown that the prevalence of CVDs sharply increases after age 65, with its prevalence doubling every 5 years (Zhao et al., 2019). With the imminent population aging, CVDs pose a significant disease burden and threaten to become a global public health crisis within the next 20 years. Heart aging presents as left ventricular diastolic dysfunction (LVDD), myocardial hypertrophy, and mild interstitial fibrosis (North & Sinclair, 2012). These age‐related changes serve as the pathological basis for various CVDs, including hypertrophic cardiomyopathy and heart failure (Zhang et al., 2022). While recent progress has provided insight into the links between the pathology of heart aging and mitochondrial dysfunction, oxidative stress, autophagy, and telomere shortening (Abdellatif et al., 2023), much remains unknown about the precise mechanisms of the pathogenesis of heart aging.

Nicotinamide adenine dinucleotide (NAD^+^) is an indispensable coenzyme that plays a crucial role in a range of redox reactions. It serves as a vital substrate for various processes as diverse as energy metabolism, gene transcription, protein modification, and DNA repair (Imai & Guarente, 2014; Ryu et al., 2015; Verdin, 2015). Disturbances in NAD^+^ metabolism have been linked to aging and various age‐related disorders (Braidy et al., 2019; López‐Otín et al., 2016). In contrast, enhancing NAD^+^ levels through interventions such as activating the NAD^+^ biosynthetic pathways, inhibiting the NAD^+^ degradation, and replenishing NAD^+^ precursors have been found to have beneficial effects against aging (López‐Otín et al., 2016; Yoshino et al., 2018). Poly (ADP‐ribose) polymerase 1 (PARP1) is a polymerase that utilizes ADP‐ribose units derived from NAD^+^ to catalyze the polymerization of ADP‐ribose onto substrates (Sukhanova et al., 2016). It participates in a wide range of biological processes (Schreiber et al., 2006), including DNA damage repair, cell differentiation, and immune response (Luo & Kraus, 2012; Matveeva et al., 2016). However, excessive activation of PARP1 results in a significant depletion of NAD^+^, leading to mitochondrial dysfunction and cell death (Zhang et al., 2019). Although the molecular function of PARP1 has been studied, research on its role in age‐related cardiac dysfunction is still limited, and the cause of its abnormal activation in senescent cardiomyocytes remains unclear. Therefore, investigating the upstream regulator of PARP1 and clarifying its regulatory pathways could be of great significance in developing prevention and treatment strategies for heart aging.

MicroRNAs (miRNAs), approximately 22 nucleotides, are highly conserved between species and repress messenger RNA (mRNA) translation by targeting the 3′ untranslated region (UTR) of mRNAs (Van Rooij & Olson, 2012). Thousands of miRNAs regulate up to 30% genes in differential spatial and temporal expression patterns, participating in various physiological and pathological processes (Hur et al., 2013; Qiu et al., 2016; Rasko & Wong, 2017). Growing evidence has demonstrated that miR‐203 regulates tumor by regulating cell proliferation, angiogenesis, and tumor metastasis (Lohcharoenkal et al., 2018; Marisetty et al., 2017; Wang et al., 2012). Furthermore, miR‐203 has been found to be involved in several cardiovascular diseases, including diabetic cardiomyopathy (DCM) (Yang et al., 2019), myocardial infarction (MI) (Wang et al., 2021) and myocardial ischemia–reperfusion (I/R) injury (Tan et al., 2020). Recently, our published studies suggested that miR‐203 plays a role in controlling obesity and dyslipidemia by inhibiting ASBT expression (Liu, Cheng, et al., 2022). These studies suggest that miR‐203 plays a critical role in regulating disease development, and may have potential as a gene therapy tool and a biomarker for disease prevention and treatment. However, it is currently unclear whether miR‐203 is involved in heart aging.

In this study, we found that the expression of miR‐203 was downregulated in the blood and heart during the aging process, resulting in reduced NAD^+^ levels due to its promotion of PARP1 expression. In contrast, miR‐203 overexpression was associated with alleviation of heart aging by maintaining NAD^+^ levels.

## 2 RESULTS

### 2.1 miR‐203 is a potential regulator of age‐related cardiac dysfunction

**FIGURE 1:**
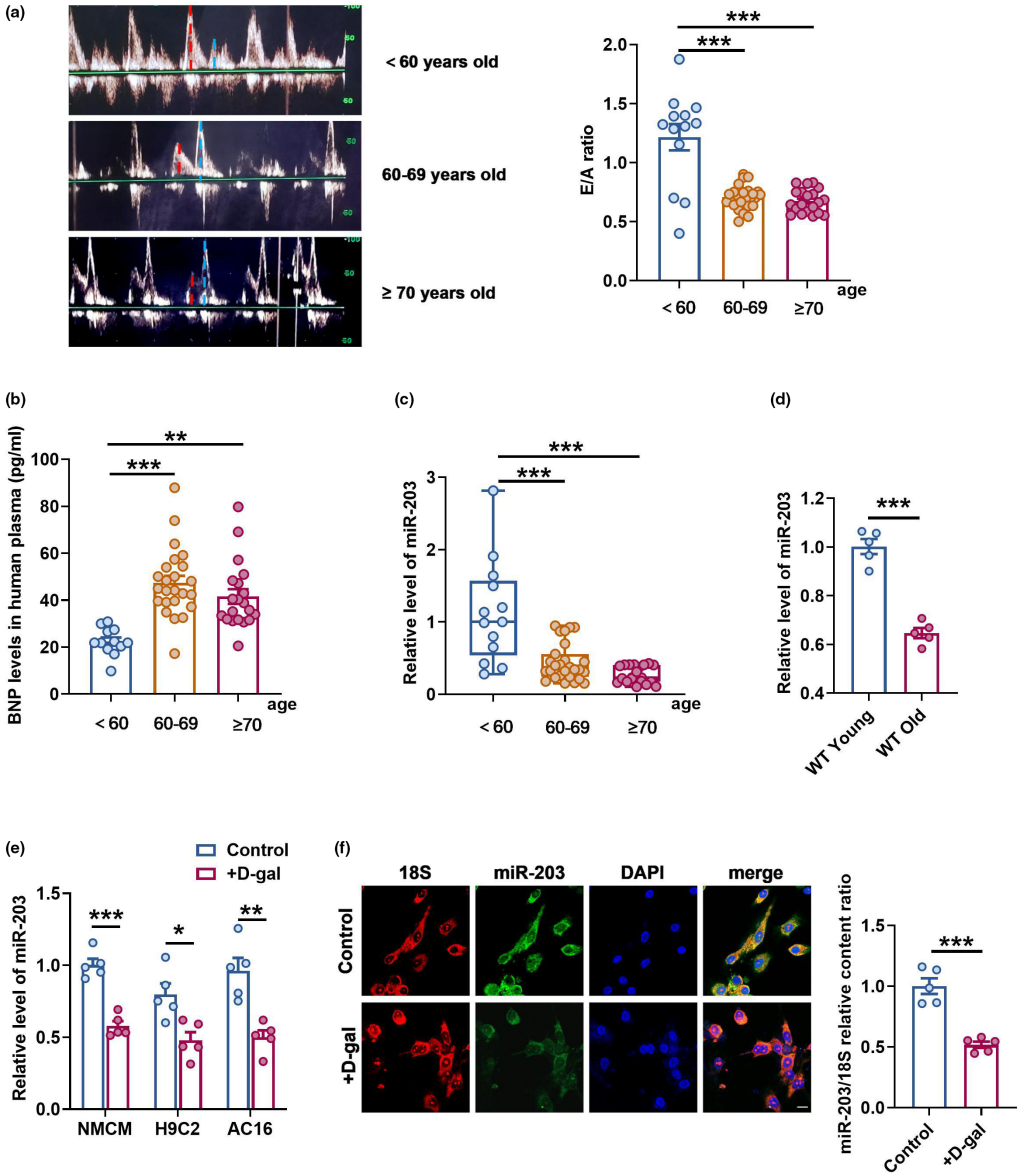
The expression level of miR‐203 in humans and mice is correlated with aging. (a) Representative echocardiograms and E/A ratio statistics in humans, showing the ratio of peak E (red dashed line) to peak A (blue dashed line) (E/A ratio) in different groups for assessing diastolic function in humans. (b) The level of B‐type natriuretic peptide in plasma was measured by Elisa. (c) The expression level of miR‐203 in blood of different groups was detected by qRT‐PCR. age <60, *n* = 13; age 60–69, *n* = 27; age ≥70, *n* = 20. (d) The expression level of miR‐203 in the hearts of young (3 months old) and old (24 months old) mice was verified by qRT‐PCR (*n* = 5 per group). (e) The expression level of miR‐203 in normal and senescent (treated with D‐gal (40 mg/mL) for 48 h) primary neonatal mice cardiomyocytes (NMCM), rat cardiomyocytes cell line (H9C2) and human cardiomyocytes cell line (AC16) was verified by qRT‐PCR (*n* = 5 per group). (f) The expression and localization of miR‐203 in normal and senescent (treated with D‐gal (40 mg/mL) for 48 h) primary cardiomyocytes verified by FISH. Scale bar, 10 μm. *p < 0.05; **p < 0.01; ***p < 0.001. Data are presented as mean ± SEM.

MiR‐203 is a stable biomarker in blood circulation that has shown potential in identifying the progression of tumor and obesity (Hur et al., 2017; Imaoka et al., 2016; Liu, Cheng, et al., 2022). To further evaluate the diagnostic value of miR‐203 in the context of aging, particularly heart aging, we included a total of 60 subjects in this study. Multiple parameters were collected and analyzed to assess their association with the relative level of miR‐203 in their blood samples. The clinical characteristics of the study population are listed in Table 1. The participants comprised a diverse range of age groups, with women constituting 65% of the individuals. As an established indicator of diastolic function, the E/A ratio exhibited a significant age‐related decrease (*p* < 0.001), with the average E/A ratio falling below one for the study population above age 60 (Table 1, Figure 1a). Furthermore, we observed an increase in plasma levels of B‐type natriuretic peptide (BNP) (*p* < 0.001), this notable change is particularly significant between subjects aged between 60 and 69 and those who were under 60 at the time (Table 1, Figure 1b). Additionally, compared with subjects under 60 years old, the expression of miR‐203 was significantly reduced in aging people (Figure 1c). To further delineate the more accurate relationship between miR‐203 and age, we performed univariate and multivariate linear regression analyses, with adjusted covariates for clinical and echocardiographic variables related to miR‐203 listed in Table 1. As demonstrated in Table 2, both univariable and multivariable analysis revealed significant negative correlations between age (*p* < 0.001 and *p* = 0.014) and BNP levels (*p* < 0.001 and *p* = 0.046) with blood miR‐203 level, and a positive correlation between E/A ratio (*p* < 0.001 and *p* = 0.002) with miR‐203 level.

**TABLE 1:** Clinical characteristics of the study population.

Subject characterization	<60 (*n* = 13)	60–69 (*n* = 27)	≥70 (*n* = 20)	*p*‐Value
Age, years	45 ± 11	65 ± 5	77.5 ± 7.5	N/A
Gender	M:5; F:8	M:9; F:18	M:7; F:13	N/A
Body mass index, kg/m^2^	31.75 ± 14.17	25.27 ± 5.55	25.88 ± 3.80	0.69
*Laboratory parameters*				
LDL cholesterol, mg/dL	3.28 ± 1.51	3.59 ± 1.81	3.32 ± 1.70	0.80
HDL cholesterol, mg/dL	1.47 ± 0.47	1.42 ± 0.66	1.71 ± 0.70	0.12
TG, mmoL/L	2.91 ± 2.14	3.47 ± 2.82	1.53 ± 0.93	0.08
TC, mmoL/L	5.03 ± 1.48	5.56 ± 2.14	4.86 ± 1.87	0.82
B‐type natriuretic peptide, pg/mL	20.33 ± 10.52	52.56 ± 35.31	50.12 ± 29.61	<0.0001
EF, %	74.00 ± 13.00	65.45 ± 5.05	65.75 ± 5.75	0.14
FS, %	35.65 ± 3.65	35.00 ± 5.00	34.00 ± 5.00	0.86
SV, mL	58.00 ± 12.00	72.50 ± 24.50	67.00 ± 23.00	0.53
E/A ratio	1.14 ± 0.74	0.92 ± 0.42	0.69 ± 0.14	<0.0001

*Note*: Values are mean ± SD, *n* (%), or median (25th to 75th percentile).

Abbreviations: A, late diastolic transmitral flow velocity; E, early diastolic transmitral flow velocity; EF, ejection fraction; FS, fractional shortening; HDL, high‐density lipoprotein; LDL, low‐density lipoprotein; SV, stroke volume; TC, total cholesterol; TG, triglyceride.

**TABLE 2:** Clinical and echocardiographic variables associated with miR‐203.

	Univariable		Multivariable	
	Sβ (95% CI)	*p*‐Value	Sβ (95% CI)	*p*‐Value
Age, years	−0.715 (−0.036 to −0.021)	<0.001	−0.330 (−0.024 to −0.003)	0.014
Gender	0.121 (−0.142 to 0.386)	0.358		
Body mass index, kg/m^2^	−0.180 (−0.055 to 0.010)	0.170		
LDL cholesterol, mg/dL	0.016 (−0.150 to 0.169)	0.905		
HDL cholesterol, mg/dL	−0.038 (−0.458 to 0.343)	0.774		
TG, mmoL/L	0.103 (−0.074 to 0.170)	0.434		
TC, mmoL/L	0.020 (−0.139 to 0.162)	0.880		
B‐type natriuretic peptide, pg/mL	−0.443 (−0.022 to −0.007)	<0.001	−0.184 (−0.012 to 0.000)	0.046
EF, %	0.208 (−0.006 to 0.052)	0.111		
FS, %	−0.084 (−0.076 to 0.039)	0.524		
SV, mL	−0.125 (−0.022 to 0.008)	0.342		
E/A ratio	0.721 (0.896 to 1.503)	<0.001	0.415 (0.270 to 1.110)	0.002

*Note*: Univariable linear regression models were constructed to identify the clinical and echocardiographic variables associated with blood miR‐203 levels. Factors with a significance level of *p* < 0.1 were selected as independent variables for the multivariable analysis. 95% confidence intervals were provided.

Abbreviations: Sβ, standardized β; other abbreviations as in Table 1.

Other than assessing miR‐203 levels in blood from the clinical study population, we also measured its expression in mouse hearts and D‐galactose (D‐gal)‐induced senescent cardiomyocytes including primary neonatal mouse cardiomyocyte (NMCM), rat cardiomyocytes cell line H9C2, and human cardiomyocytes cell line AC16 (Figure S1a). Our results revealed a significant reduction in miR‐203 levels in the hearts of wild type (WT) Old mice (24 months old) compared to those of WT Young mice (3 months old), as well as in senescent cardiomyocytes induced by D‐gal (Figure 1d,e). Furthermore, fluorescence in situ hybridization (FISH) analysis in Figure 1f also determined the primary cytoplasmic localization of miR‐203, indicating its potential regulatory effects within cytoplasm in senescent cardiomyocytes. These findings imply that miR‐203 may serve as a reliable blood biomarker for monitoring heart aging and may also possess a potential regulatory role in the aging process.

### 2.2 Overexpression of miR‐203 attenuates cardiac dysfunction in natural aging mice

**FIGURE 2:**
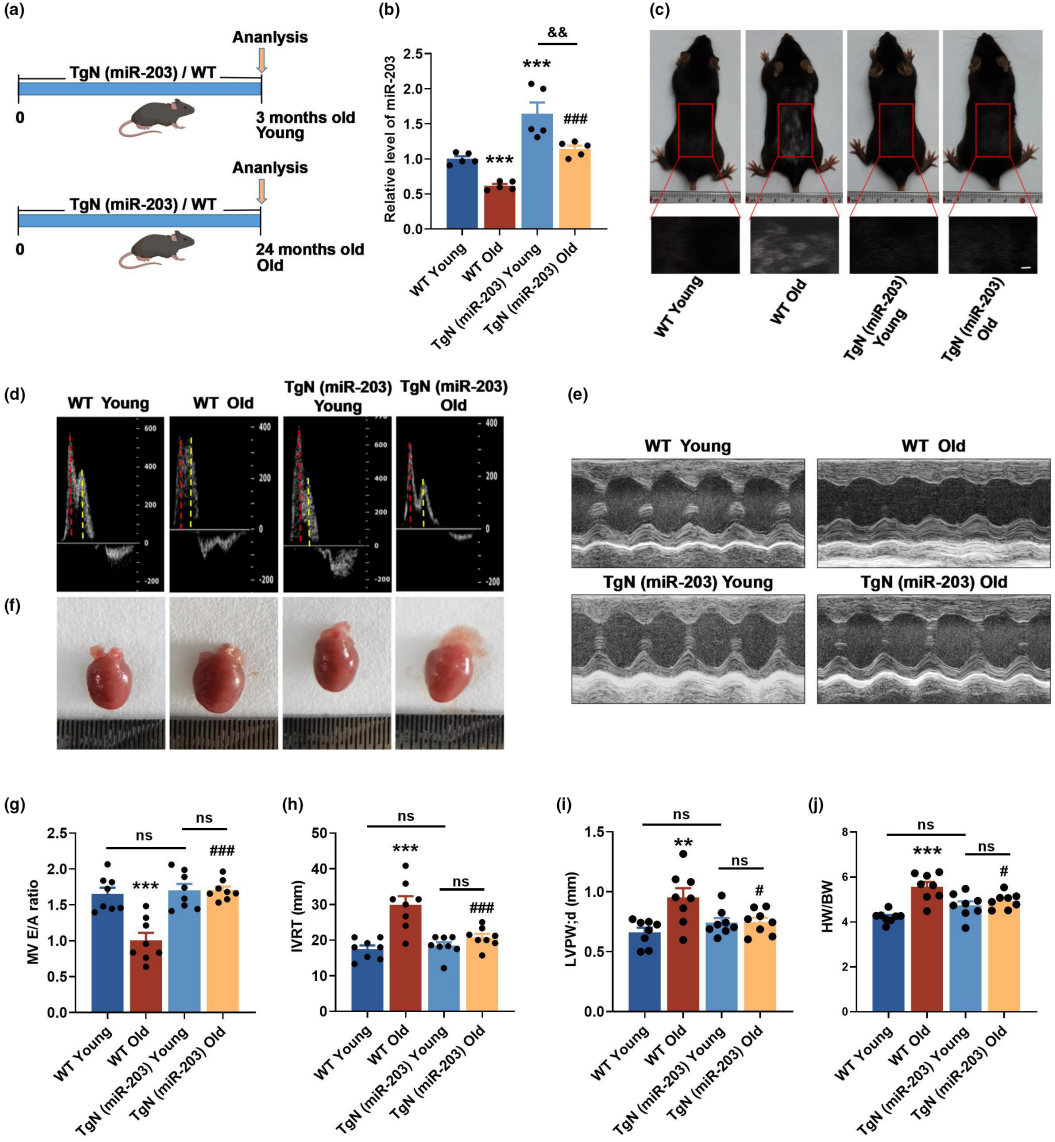
Overexpression of miR‐203 reduces cardiac diastolic dysfunction in aging mice. (a) Schematic diagram of the in vivo experimental procedure in mice. Both WT and TgN (miR‐203) mice were used, consisting of young mice at 3 months of age and naturally aging mice at 24 months of age. (b) qRT‐PCR analysis of miR‐203 overexpression efficiency in the myocardium of young (3 months old) and aging (24 months old) mice (*n* = 5 per group). ***p < 0.001 vs WT Young, ^###^ *p* < 0.001 vs WT Old, ^&&^ *p* < 0.01. (c) Photos of the back hair of mice in each group. Red boxes indicate areas of exposed hair loss skin (*n* = 5 per group). Scale bar, 4 mm. (d) Representative echocardiograms of mice in each group, showing the effect of overexpression of miR‐203 on the ratio of peak E (red dashed line) to peak A (yellow dashed line) (E/A ratio) for assessing cardiac diastolic function in aging mice (*n* = 8 per group). (e) Representative M‐mode echocardiograms of mice in each group. (f) Representative heart photographs of each group of mice. (g) Statistical results of echocardiographic E/A ratio of mice in each group (*n* = 8 per group). (h) Isovolumetric relaxation time (IVRT) statistics. *n* = 8. ***p < 0.001 vs WT Young, ^###^ *p* < 0.001 vs WT Old. (i) Left ventricular posterior wall end‐diastolic thickness (LVPW; d) statistics. *n* = 8. **p < 0.01 vs WT Young, ^#^ *p* < 0.05 vs WT Old. (j) Statistical results of the ratio of heart weight to body weight (HW/BW) of mice in each group. *n* = 8. ***p < 0.001 vs WT Young, ^#^ *p* < 0.05 vs WT Old. ns, both ends of the horizontal line are not significant. Data are presented as mean ± SEM.

To explore whether miR‐203 provides beneficial effects on the aging heart, we constructed transgenic mice with systemic overexpression of miR‐203, and then compared cardiac function in WT and TgN (miR‐203) mice at different ages (Figure 2a). The qRT‐PCR results revealed a significant down‐regulation of miR‐203 expression in the hearts of old WT mice compared to young ones. Compared with WT mice of the same age, young TgN (miR‐203) mice had significantly elevated levels of miR‐203. Even with overexpression, miR‐203 decreased with age. However, in aged TgN mice, the decline in miR‐203 levels was noticeably less significant (Figure 2b). Upon visual inspection, TgN (miR‐203) Old mice appeared to have denser and smoother hair than WT Old mice (Figure 2c). Transthoracic echocardiography was also performed to evaluate the cardiac function of the two different groups of mice at different ages. The results from the diastolic function‐associated parameters, including the early‐to‐late transmitral filling velocity (E/A) ratio and isovolumic relaxation time (IVRT), indicated that TgN (miR‐203) Old mice exhibited better heart functionalities compared to WT Old mice (Figure 2d,g,h). As measured, the increase of left ventricular posterior wall (LVPW) thickness at the end of the diastolic period and heart weight/body weight (HW/BW) ratio suggested cardiac hypertrophy in WT Old mice, while these measurements remained at lower levels in the hearts of TgN (miR‐203) Old mice (Figure 2e,i,j). As shown in Figure 2f, the hearts of WT Old mice showed clear hypertrophy compared with those of WT Young mice, while the hearts of TgN (miR‐203) Old mice showed no significant changes compared with those of Young mice. We then examined ANP and BNP levels in the hearts of WT and TgN (miR‐203) mice at different ages (Figure S1b,c). Compared with WT Young mice, ANP and BNP levels were significantly increased in the hearts of WT Old mice. These results are consistent with other published studies (Charloux et al., 2008; Chen, Zhou, et al., 2022). Moreover, the ANP and BNP levels were remarkably decreased in TgN (miR‐203) Old mice. Additionally, there were no notable aberrations in systolic function observed in aging mice when compared to their younger counterparts. This suggests the presence of heart failure with preserved ejection fraction (HFpEF) in WT Old mice (Figure S1d,e). Overall, these findings indicate that miR‐203 has the potential to attenuate cardiac diastolic dysfunction in aging mice.

### 2.3 Overexpression of miR‐203 mitigates cardiac senescence in natural aging mice

**FIGURE 3:**
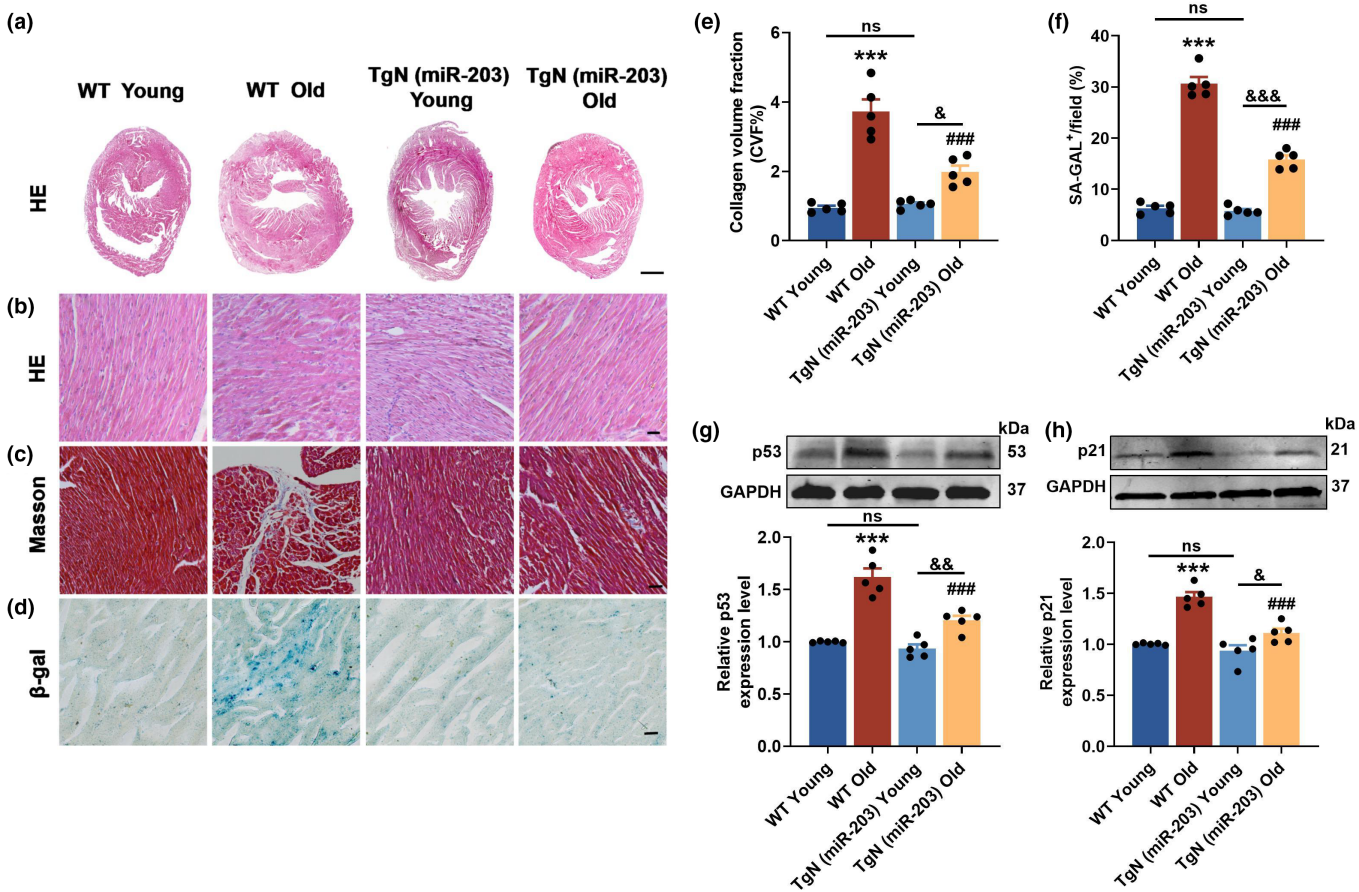
Overexpression of miR‐203 reduces cardiac remodeling and delays heart aging in aging mice. (a) Hematoxylin and eosin (HE) staining of transverse section of whole heart. Scale bar, 600 μm. (b and c) Hematoxylin and eosin (HE) staining (b) and Masson trichromatic staining (c) of transverse section of zoomed‐in areas (×20) (*n* = 5 per group). Scale bar, 20 μm. (d) β‐gal staining of mouse heart tissue sections (×20) (*n* = 5 per group). Scale bar, 20 μm. (e) Statistical analysis of collagen in (c). *n* = 5. ***p < 0.001 vs WT Young, ^###^ *p* < 0.001 vs WT Old. (f) Statistical analysis of the proportion of β‐gal positive areas in mouse heart tissue slices. *n* = 5. ***p < 0.001 vs WT Young, ^###^ *p* < 0.001 vs WT Old. (g and h) Western blot analysis of protein expression and relative level of p53 (g) and p21 (h) in mouse hearts (*n* = 5 per group). GAPDH served as an internal control. ***p < 0.001 vs WT Young, ^###^ *p* < 0.001 vs WT Old. ^&^ *p* < 0.05; ^&&^ *p* < 0.01; ^&&&^ *p* < 0.001. ns, both ends of the horizontal line are not significant. Data are presented as mean ± SEM.

Myocardial morphology and collagen deposition were analyzed by HE staining and Masson staining, respectively. In contrast to WT Young mice, WT Old mice exhibited myocardial fiber disarrangement and collagen deposition, which were remarkably reduced in TgN (miR‐203) Old mice (Figure 3a–c,e). To assess cellular senescence in cardiac tissue, we performed senescence‐associated β‐galactosidase (SA‐β‐Gal) staining. The results showed increased positive staining of SA‐β‐Gal in hearts of WT Old mice, whereas staining was less prominent in TgN (miR‐203) Old mice (Figure 3d,f). Moreover, the down‐regulation of senescence markers p53 and p21 further supported the beneficial effects of miR‐203 on cardiac senescence (Figure 3g,h). Taken together, these results illustrate that miR‐203 holds promise for mitigating cardiac remodeling and senescence in aging mice.

### 2.4 Gain and loss of miR‐203 function influence cardiomyocyte senescence

**FIGURE 4:**
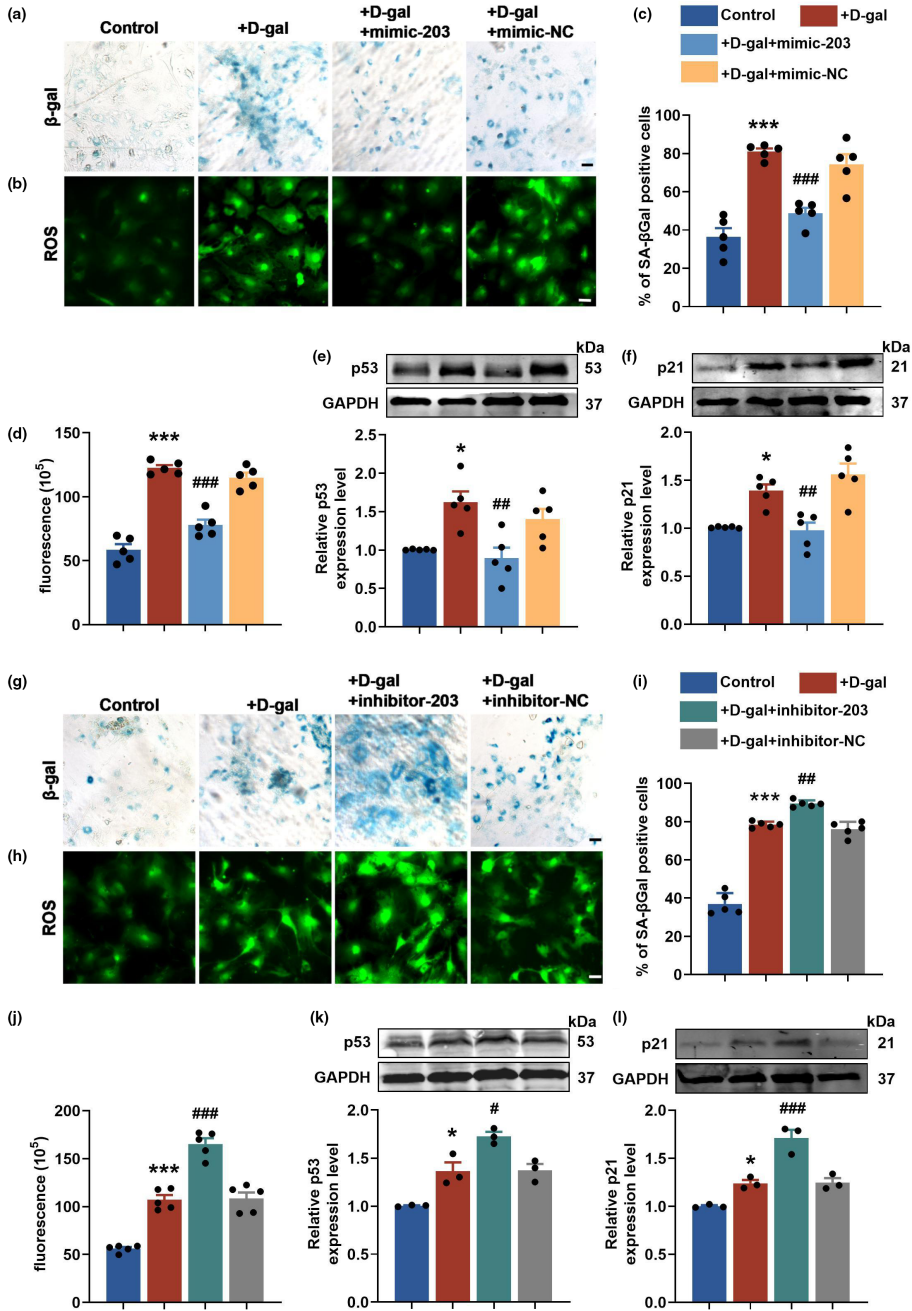
Gain and loss of miR‐203 function exert opposing effects on cardiomyocyte senescence. (a and b) Representative images (×20) of β‐gal staining (a) and ROS staining (b) (*n* = 5 per group). Primary cardiomyocytes transfected with mimic‐203 or negative control (NC) were treated with D‐gal (40 mg/mL) for 48 h. Scale bar, 20 μm. (c and d) Statistical analysis of the percentage of β‐gal positive cells (c) and fluorescence intensity of ROS images (d). *n* = 5. (e and f) Western blot analysis of protein expression and relative level of p53 (e) and p21 (f) in cardiomyocytes (*n* = 5 per group). (g and h) Representative images (×20) of β‐gal staining (g) and ROS staining (h) (*n* = 5 per group). Primary cardiomyocytes transfected with inhibitor‐203 or NC were treated with D‐gal (40 mg/mL) for 48 h. Scale bar, 20 μm. (i and j) Statistical analysis of the percentage of β‐gal positive cells (i) and fluorescence intensity of ROS images (j). *n* = 5. (k and l) Western blot analysis of protein expression and relative level of p53 (k) and p21 (l) in cardiomyocytes (*n* = 3 per group). GAPDH served as an internal control. *p < 0.05; ***p < 0.001 vs control, ^#^ *p* < 0.05; ^##^ *p* < 0.01; ^###^ *p* < 0.001 vs D‐gal. Data are presented as mean ± SEM.

D‐gal was utilized to induce senescence in cardiomyocytes, resulting in a noticeable rise in the number of SA‐β‐Gal positive cells. To counteract this effect, exogenous miR‐203 mimic (mimic‐203) was transfected to foster miR‐203 expression (Figure S2a), which led to a notable decrease in senescence caused by D‐gal (Figure 4a,c). Since the accumulation of reactive oxygen species (ROS) is one of the main features of senescent cells, we conducted experiments to assess the levels of ROS in cardiomyocytes. The results depicted in Figure 4b,d demonstrated that mimic‐203 effectively diminished the excessive accumulation of ROS in senescent cardiomyocytes. The anti‐senescence effects of miR‐203 were further confirmed by a significant decline in the expression of two senescence‐associated proteins, p53 and p21, following treatment with mimic‐203, as illustrated in Figure 4e,f. On the contrary, transfection of senescent cardiomyocytes with a miR‐203 inhibitor (inhibitor‐203) to inhibit endogenous miR‐203 level brought about an increase in the percentage of SA‐β‐Gal positive cells, ROS levels, and p53 and p21 protein levels (Figure 4g–l and Figure S2b). These results provide concrete evidence of the regulatory capacity of miR‐203 in cardiomyocyte senescence.

### 2.5 miR‐203 targets 3′UTR of PARP1 mRNA to restore NAD^+^ levels and mitochondrial function in heart aging

**FIGURE 5:**
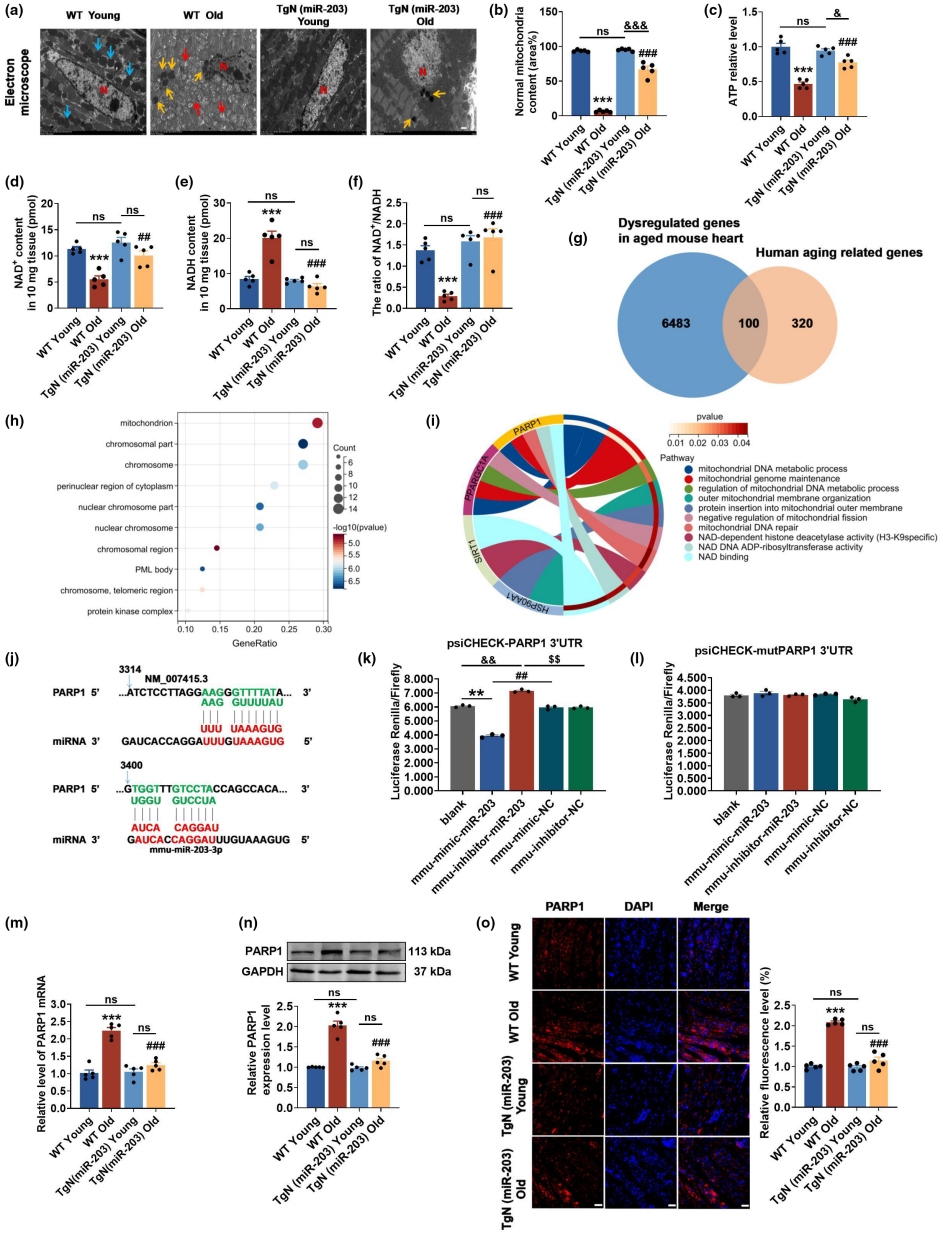
miR‐203 restores NAD^+^ levels and improves mitochondrial function by inhibiting the expression of NAD^+^ consuming enzyme PARP1. (a) Representative electron microscopic images (×2 k) of mouse heart tissue sections (*n* = 5 per group). The blue arrow represents normal mitochondria, red arrow represents swollen mitochondria, yellow arrow represents lipofuscin, and red N represents the nucleus. Scale bar, 10 μm. (b–f) Statistical analysis of the relative number of normal mitochondria in electron microscope images (b), ATP content (c), NAD^+^ content (d), NADH content (e), and NAD^+^/NADH ratio (f) in mouse myocardial tissue. ***p < 0.001 vs WT Young, ^##^ *p* < 0.01; ^###^ *p* < 0.001 vs WT Old, ^&^ *p* < 0.05; ^&&&^ *p* < 0.001. (g) Common genes intersected between dysregulated genes in aged mouse hearts and human aging‐related genes. (h) GO enrichment analysis of genes in Figure (g), among which the Cellular Component was mainly enriched in mitochondria. (i) Circos plot of mitochondrial‐related genes and pathways. (j) Complementary sequences between miR‐203 (red font) and PARP1 (green font) predicted by RNAhybrid database. (k and l) The detection of miR‐203 binding to wild type 3′UTR of PARP1 and the mutant 3′UTR of PARP1 by luciferase reporter gene (*n* = 3 per group). (m) qRT‐PCR analysis of PARP1 mRNA levels in the myocardium of young (3 months old) and aging (24 months old) mice (*n* = 5 per group). (n) Western blot analysis of PARP1 protein expression and relative levels in mouse heart tissues (*n* = 5 per group). (o) Representative immunofluorescence image (×20) of PARP1 (red) in mouse heart tissue, with nucleus labeled by DAPI staining (blue). Scale bar, 20 μm (*n* = 5 per group). ***p < 0.001 vs WT Young, ^###^ *p* < 0.001 vs WT Old. ns, both ends of the horizontal line are not significant. Data are presented as mean ± SEM.

In‐depth exploration of the specific regulatory mechanism of miR‐203 in heart aging, we utilized transmission electron microscopy (TEM) to observe the ultrastructural changes in heart tissue. As presented in Figure 5a,b, there was clear cardiac mitochondrial damage in WT Old mice, characterized by mitochondrial swelling and loss of intact structure, as well as lipofuscin accumulation. In contrast, TgN (miR‐203) Old mice exhibited mitochondrial morphology and size much closer to its young counterparts, indicating the beneficial impact of miR‐203 on the mitochondrial health. Given the significance of energy metabolism in supporting optimal mitochondrial function, we investigated intracellular ATP production and NAD^+^/NADH levels. The outcomes served as a measure of mitochondrial activity and function. Our findings demonstrated that ATP levels, NAD^+^ levels, and the NAD^+^/NADH ratio were considerably reduced in WT Old mice, while these reductions were not as significant in TgN (miR‐203) Old mice (Figure 5c–f). In order to pinpoint the downstream targets controlled by miR‐203 that specifically influence mitochondrial function, we employed bioinformatics methods for data mining. Dysregulated genes in heart tissue of aging mice from the GEO database (GSE72890) and human aging‐related genes from the Aging Atlas database were utilized for analysis, resulting in the identification of 100 common genes, as demonstrated in Figure 5g. Further GO analysis revealed that the majority of these genes were related to the mitochondria (Figure 5h). Taking into consideration the potential protective effect of miR‐203 on mitochondrial energy metabolism and NAD^+^ functions, we utilized Circos plot analysis and identified four genes linked to these two biological processes (Figure 5i). Among these genes, mimic‐203 exhibited the most substantial reduction in PARP1 mRNA levels (Figure S3a). Additionally, the minimum free energy associated with the binding of miR‐203 to PARP1 mRNA was found to be the lowest (Table S1). PARP1 is considered the primary consumer of NAD^+^ in the nucleus, and the excessive depletion of the NAD^+^ pool (50%–80%) by PARP1 in response to DNA damage has significant implications for mitochondria and leads to ATP depletion (Rudolph et al., 2021). After employing the RNAhybrid prediction method, we were able to identify a binding sequence for miR‐203‐3p located in the 3′UTR of PARP1 mRNA (Figure 5j). Dual‐luciferase reporter assay further confirmed the binding of miR‐203 to the 3′UTR of PARP1 (Figure 5k,l). Additionally, we evaluated PARP1 expression in the hearts of mice from different groups, the results showed that PARP1 expression was significantly upregulated in WT Old mice comparing to WT Young mice, whereas decreased levels were observed in TgN (miR‐203) Old mice (Figure 5m–o). Consistently, the mRNA levels of PARP1 were significantly increased in human blood samples from individuals aged over 60 years (Figure S3b).

### 2.6 Overexpression of miR‐203 improved mitochondrial function in senescent cardiomyocytes

**FIGURE 6:**
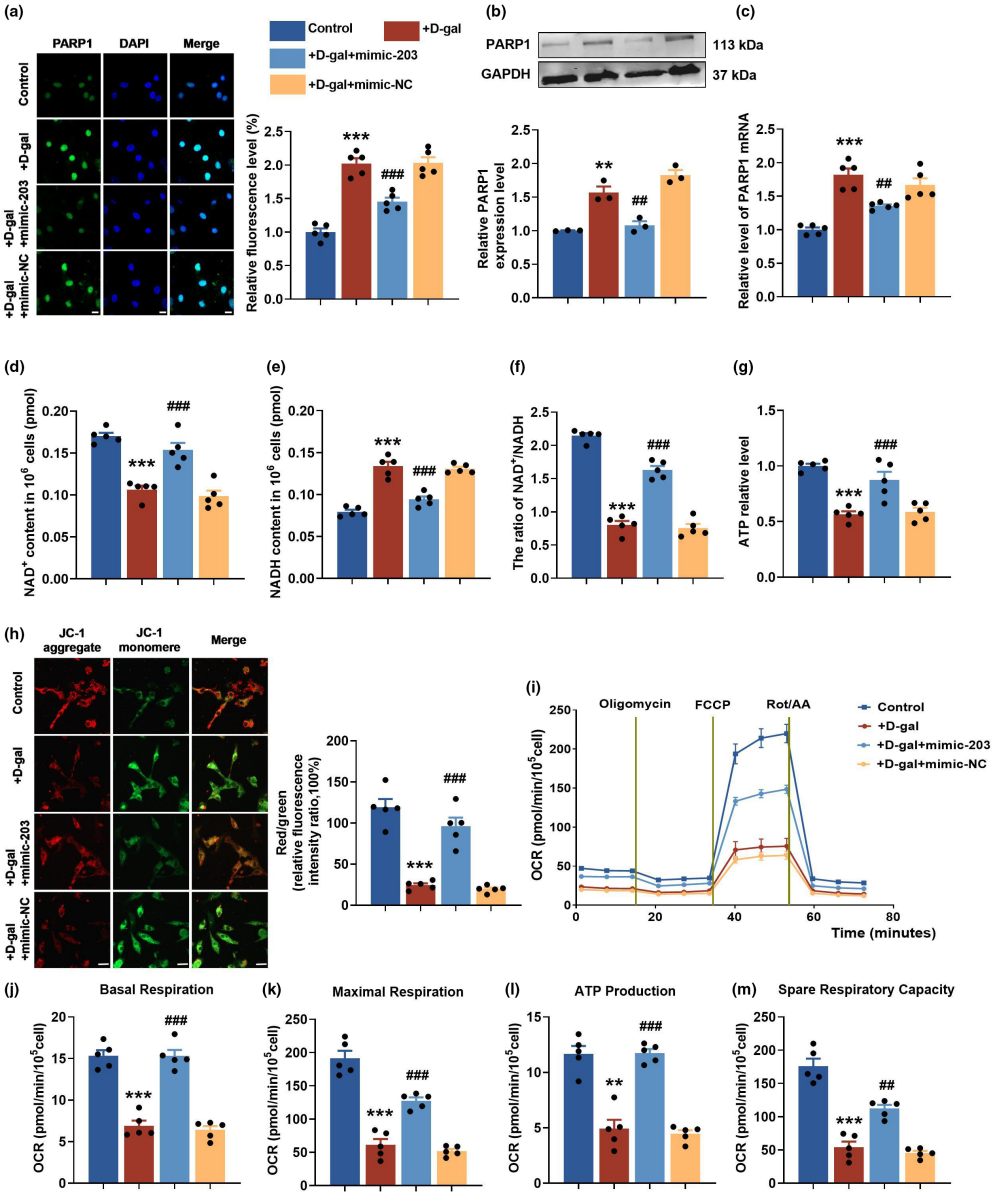
Overexpression of miR‐203 restores NAD^+^ levels and improves mitochondrial function in cardiomyocytes. (a) Representative images (left) and fluorescence intensity statistics (right) of PARP1 (green) immunofluorescence (×63) in cardiomyocytes transfected with or without mimic‐203 and treated with D‐gal (40 mg/mL) for 48 h. Scale, 10 μm. (b) Western blot analysis of PARP1 protein expression and relative level statistics in cardiomyocytes transfected with or without mimic‐203 and treated with D‐gal (*n* = 3 per group). (c) qRT‐PCR analysis of PARP1 mRNA levels in cardiomyocytes transfected with or without mimic‐203 and treated with D‐gal. (d–g) Statistical analysis of NAD^+^ content (d), NADH content (e), NAD^+^/NADH ratio (f), and ATP levels (g) in primary cardiomyocytes transfected with or without mimic‐203 and treated with D‐gal. (*n* = 5 per group). (h) Representative images (left) and fluorescence intensity statistics (right) of cardiomyocyte JC‐1 staining (×40) (*n* = 5 per group). Scale, 20 μm. (i) Measurement of cardiomyocyte oxygen consumption rate (OCR) by Seahorse XFe96 analyzer (*n* = 5 per group). (j–m) Statistical analysis of basal respiration (j), maximal respiration (k), ATP production (l), and spare respiratory capacity (m) in cardiomyocytes. **p < 0.01; ***p < 0.001 vs control, ^##^ *p* < 0.01; ^###^ *p* < 0.001 vs D‐gal. Data are presented as mean ± SEM.

In vitro, we conducted further investigations to reveal the potential protective effect of miR‐203 on mitochondrial function by targeting PARP1 in senescent cardiomyocytes. Using immunofluorescence and western blot techniques, we observed that exogenous transfection of miR‐203 could reduce PARP1 protein levels raised by D‐gal (Figure 6a,b). This finding was further confirmed by qRT‐PCR, which showed that transfection with mimic‐203 effectively decreased the elevated PARP1 mRNA level induced by D‐gal (Figure 6c). On top of that, the results from mitochondrial function evaluations revealed that treatment with D‐gal led to a decrease in the NAD^+^, NAD^+^/NADH ratio, and ATP levels, but an increase in NADH level. However, overexpression of miR‐203 significantly reverted these effects (Figure 6d–g). Mitochondrial membrane potential was measured using JC‐1 dye, and the potential recovered significantly in senescent cardiomyocytes transfected with miR‐203 mimic, as manifested by the increased fluorescence intensity ratio of JC‐1 aggregate to JC‐1 monomer (Figure 6h). Consistent with the results above, overexpression of miR‐203 has been shown to significantly enhance mitochondrial respiration in senescent cardiomyocytes, including measurements of basal respiration, maximum respiration, ATP production, and residual respiration (Figure 6i–m).

Furthermore, transfection of PARP1 overexpression plasmid (oe‐PARP1) in primary cardiomyocytes led to an increase in PARP1 mRNA levels and induced cardiomyocyte senescence. This was evidenced by an elevated number of β‐gal staining positive cells, increased levels of ROS, decreased NAD^+^ content, reduced NAD^+^/NADH ratio, and disturbance of mitochondrial membrane potential. Notably, co‐transfection of mimic‐203 significantly decreased the mRNA levels of PARP1 and improved the senescence phenotype of cardiomyocytes induced by PARP1 overexpression (Figure S4).

### 2.7 Knockdown of miR‐203 aggravated mitochondrial dysfunction of senescent cardiomyocytes

We then sought to determine the effects of miR‐203 knockdown on mitochondrial function in cardiomyocytes undergoing senescence. As anticipated, reduction of miR‐203 via transfection of inhibitor‐203 caused an increase in PARP1 expression (Figure S5a,b). In addition, D‐gal‐induced senescent cardiomyocytes transfected with inhibitor‐203 exhibited a reduction in NAD^+^ levels, NAD^+^/NADH ratio, and ATP levels, along with an increase in NADH levels (Figure S5c–f). Moreover, the knockdown of miR‐203 significantly impaired the mitochondrial membrane potential and mitochondrial respiration, which suggests a further decline in mitochondrial function due to inhibitor‐203 treatment (Figure S5g–l).

## 3 DISCUSSION

Aging is a major risk factor for cardiovascular disease, which is the leading cause of death worldwide. As the global population continues to age, age‐related cardiovascular disease is expected to become a new public health challenge. In this study, we elucidated the role and mechanism of miR‐203 in regulating age‐related cardiac dysfunction. By constructing miR‐203 overexpression transgenic mice and conducting in vitro experiments, we demonstrated that miR‐203 overexpression effectively ameliorates cardiac diastolic dysfunction in naturally aging mice, and inhibits cardiac structural remodeling and cardiomyocyte injury. Mechanistically, miR‐203 reduces PARP1 expression by directly binding to the 3′UTR of PARP1 mRNA, increasing the level of NAD^+^ and improving energy metabolism and mitochondrial damage, thereby inhibiting cardiomyocyte senescence. This study reveals a new pathogenesis of heart aging and provides a reliable drug target for prediction and treatment of aging‐related cardiac dysfunction.

Non‐coding RNAs, especially miRNAs, have emerged as critical regulators and predictors of various diseases. With the advancement of small nucleic acid drugs and precision medicine, their clinical potential is increasingly recognized. Notably, extracellular miRNAs, in particular, offer great promise as disease biomarkers due to their stability and non‐invasive detection in various body fluids (Mori et al., 2019). Keun Hur et al. and Hiroki Imaoka et al. have revealed the potential of plasma miR‐203 as a biomarker for predicting metastasis and poor prognosis in human colorectal and gastric cancer, respectively (Hur et al., 2017; Imaoka et al., 2016). Our previous research has shown that plasma miR‐203 levels are modifiable in individuals with varying BMI, suggesting its utility as a plasma marker for the early prediction of obesity (Liu, Cheng, et al., 2022). In this study, we assessed the association between aging, cardiac performance, and miR‐203 level in a general population sample without prevalent cardiovascular risk factors and structural heart disease. Our findings reveal a significant decrease in miR‐203 levels in the blood of individuals aged 60 years and older compared to those younger than 60 years of age, indicating a negative correlation between miR‐203 levels and age. Notably, we observed that in participants with normal left ventricular ejection fraction (LVEF), the E/A ratio decreased with increasing age (Ishida et al., 2021), while B‐type natriuretic peptide (BNP) levels were elevated. These observations align with the criteria outlined in the 2021 ESC Guidelines for the diagnosis and treatment of acute and chronic heart failure, which define heart failure with preserved ejection fraction (HFpEF) (McDonagh et al., 2021). Furthermore, our findings are consistent with Yoshida et al., who suggested that increased BNP levels serve as markers of heightened risk for heart failure in older adults (Yoshida et al., 2019). Importantly, we also identified an association between miR‐203 levels, E/A ratio, and BNP, suggesting the potential utility of miR‐203 as an early indicator of left ventricular diastolic dysfunction, or possibly even HFpEF. However, further longitudinal studies with larger sample sizes are necessary to validate our findings.

To quest the regulatory role of miR‐203 in natural aging, we generated TgN (miR‐203) mice with systemic overexpression of miR‐203. Based on the results of this study and our previously published findings, there were no significant physiological differences between TgN (miR‐203) mice and WT mice, including body weight, blood lipids, and cardiac function. C57BL/6 mice and TgN (miR‐203) mice were subjected to a 24‐month feeding regimen as natural aging models. WT Old mice have shown diastolic dysfunction, myocardial fiber disturbances, collagen deposition, and cardiac senescence, which were consistent with our previously published work as well as the results of Hu et al (Hu et al., 2022; Liu, Bai, et al., 2022). Our findings revealed that TgN (miR‐203) Old mice were resistant to age‐induced diastolic dysfunction and cardiac remodeling, while no substantial alteration in systolic function was observed.

Over the past decade, research into senolytics has made significant progress, leading to a deeper understanding of their potential benefits. Senolytic therapy, including Dasatinib, Quercetin, miR‐34a, and miR‐570, shows promise in targeting senescent cells and has demonstrated effectiveness in delaying aging, preventing age‐related diseases, promoting tissue regeneration, improving functional outcomes, and even synergizing with other treatments (Barnes et al., 2019; Di Micco et al., 2021). Consequently, the targeted approach of addressing cardiomyocyte senescence emerges as a promising strategy in dealing with heart aging. In our study, TgN (miR‐203) Old mice exhibited reduced SA‐β‐gal staining and downregulation of senescence markers p53 and p21, providing evidence of the beneficial effects of miR‐203 on cardiac senescence. D‐gal is a widely recognized tool in aging research (Azman & Zakaria, 2019) and has been extensively utilized to induce cell senescence in various cell types, including neural stem cells (Cheng et al., 2019), cardiomyocytes (Wang et al., 2022), etc. A final concentration of 40 mg/mL of D‐gal for 48 h effectively induces cell senescence (Du et al., 2021; Pan et al., 2021). Transfection of exogenous mimic‐203 effectively mitigated D‐gal‐induced senescence in cardiomyocytes, as evidenced by reduced SA‐β‐gal staining, decreased ROS accumulation, and downregulated expression of senescence‐associated proteins p53 and p21. These results indicate the potential of mimic‐203 as a gene‐based senolytics for the treatment of heart aging.

Heart aging is characterized by disturbances in mitochondrial dynamics (fission/fusion), impaired mitochondrial function, increased oxidative stress, mitochondrial damage, and altered mitochondrial autophagy (Chen, Lee, & Garbern, 2022; Shirakabe et al., 2016; Xie et al., 2023). We also observed diffuse mitochondrial damage, including mitochondrial swelling, loss of intact structure, and accumulation of lipofuscin in the aging mouse heart (Li et al., 2022; Liu, Bai, et al., 2022; Wang et al., 2023). Restoring mitochondrial function has emerged as a potential therapeutic strategy for addressing age‐related cardiac dysfunction (Xie et al., 2023). Zhu et al. propose precise PGC1α regulation to restore mitochondrial homeostasis in maintaining cardiac function (Zhu et al., 2019). Additionally, Wang et al. showed that Shank3 ablation activates CaMKII/Parkin‐mediated mitophagy, preserving cardiac function during aging (Y. Wang et al., 2022). Notably, TgN (miR‐203) Old mice exhibited preserved mitochondrial morphology and size, suggesting the beneficial effects of miR‐203 on mitochondrial health. Moreover, our in vitro experiments further supported the role of miR‐203 in ameliorating D‐gal‐induced mitochondrial dysfunction in senescent cardiomyocytes. These findings confirmed that miR‐203 ameliorated heart aging by restoring mitochondrial function.

NAD^+^ levels decline with aging across various tissues (Castro‐Portuguez & Sutphin, 2020), including the aging heart (Pillai et al., 2021), and are considered an important contributor to several age‐related diseases. Previous studies have unequivocally established the remarkable effectiveness of NAD^+^ precursor supplementation, specifically nicotinamide mononucleotide (NMN) and nicotinamide riboside (NR), in combating the complex process of aging (Levine et al., 2020; Yoshino et al., 2018). Building upon these findings, Abdellatif et al. demonstrated that diastolic dysfunction in 24‐month‐old mice was significantly mitigated through the administration of the NAD^+^ precursor nicotinamide (NAM) (Abdellatif et al., 2021). Moreover, in a groundbreaking research by Yang et al. demonstrates that trace amounts of nicotine can induce NAD^+^ biogenesis, enhance glucose homeostasis and cognitive function, and mitigate the manifestations of aging (Yang et al., 2023). Our study showed a substantial decline in NAD^+^ levels in both WT Old mice and senescent cardiomyocytes, whereas NAD^+^ levels were restored in TgN (miR‐203) Old mice or senescent cardiomyocytes transfected with mimic‐203. We identified miR‐203 as a regulator of PARP1 through biogenic analysis and confirmed their interaction using a dual‐luciferase reporting assay. PARP1 depletes NAD^+^ and contributes to mitochondrial ATP depletion in response to DNA damage. Zhang et al. employed PARP1 inhibitors to mitigate NAD^+^ depletion, oxidative stress, and DNA damage in cardiomyocytes associated with atrial fibrillation (Zhang et al., 2019), while Zha et al. demonstrated that the PARP1 inhibitor PJ34 enhances the function of aging‐induced endothelial progenitor cells by maintaining intracellular NAD^+^ levels and boosting SIRT1 activity (Zha et al., 2018). Recent research conducted by Guo et al. has provided evidence that the inhibition of PARP1 enhances the activity of AMP‐activated protein kinase alpha (AMPKα) and promotes mitochondrial turnover, ultimately leading to an extension of lifespan (Guo et al., 2023). Notably, we observed a significant upregulation of PARP1 expression in WT Old mice, while decreased levels were observed in TgN (miR‐203) Old mice. These findings highlight PARP1 as a potential target for treating aging‐related dysfunction, and miR‐203 as a genetic tool for PARP1 inhibition.

In conclusion, this study demonstrates the potential of miR‐203 in delaying age‐related cardiac dysfunction. The mechanism underlying this effect is the inhibition of PARP1 overexpression in cardiomyocytes, leading to increased levels of NAD^+^ and the NAD^+^/NADH ratio. These changes ultimately improve mitochondrial energy metabolism and cardiomyocytes senescence. The findings of this study offer new insights into improving age‐related cardiac dysfunction and may identify effective drug targets and clinical therapies for cardiovascular diseases in the future.

## 4 EXPERIMENTAL PROCEDURES

### 4.1 Study approval

This study protocol has been approved by the Ethics and Science Committee of Harbin Medical University (HMU IRB1013820). Written informed consent was obtained from all enrolled subjects. Experimental procedures were conducted in accordance with the Guidelines for the Care and Use of Laboratory Animals published by the National Institutes of Health (NIH publication No. 85e23, revised 1996).

### 4.2 Human sample

A total of 120 young and elderly individuals underwent physical examination at the Second Affiliated Hospital of Harbin Medical University. Participants were excluded if they met any of the following criteria: hypertension (systolic blood pressure (SBP) ≥ 140 mmHg or diastolic blood pressure (DBP) ≥ 90 mmHg) or taking antihypertensive drugs; suffering from cardiovascular and cerebrovascular diseases (coronary artery disease, myocardial infarction, ischemic stroke, arrhythmia, cerebral hemorrhage, or heart failure, left ventricular EF < 50%); overweight and obesity (BMI ≥25 kg/m^2^); chronic obstructive pulmonary disease (COPD) with a ratio of forced expiratory volume in the first second and forced vital capacity <0.7. Following the application of these exclusion criteria, the final population for this study consisted of 60 participants without prevalent cardiovascular risk factors or structural heart disease. The clinical patient information of human blood samples is presented in Table 1. Patients signed a written informed consent form before participating.

### 4.3 MiR‐203 overexpression transgenic mice

The method for overexpressing miR‐203 in transgenic mice (TgN mice) was described in Guo et al. In brief, recombinant DNA technology was utilized to construct a vector containing the pre‐miR‐203 sequence. The pre‐miR‐203 DNA fragments were prepared by enzyme digestion, separation, and purification. The eggs of coitus‐mated female mice (C57BL/6, 2 months, 20 ± 3 g) were microinjected with DNA fragments, and the injected eggs were implanted into female mice with false pregnancies. Genomic DNA was isolated and the successful presence of the transgene was detected by PCR analysis. Wild type (WT) C57BL/6 female mice and TgN (miR‐203) female mice (20 ± 3 g) were randomly selected and divided into four groups: Young Wild type mice (WT Young), Aged Wild type mice (WT Old), Young miR‐203 overexpression transgenic mice (TgN (miR‐203) Young), and Aged miR‐203 overexpression transgenic mice (TgN (miR‐203) Old). When the young mice were 3 months old and the old mice were naturally fed up to 24 months old, Doppler ultrasound was used to detect the cardiac function of each group of mice, and photographs were taken. The hearts of mice in each group were separated and weighed, and blood from the heart and abdominal aorta were collected for further experiments.

### 4.4 Primary culture of neonatal mice cardiomyocytes

Cardiomyocytes were isolated from neonatal Kunming mice aged 1 to 3 days old, following the procedure described in our previous study. Briefly, after dissection and washing, the heart was minced and digested with 0.25% trypsin. The resulting cell suspension was centrifuged, resuspended in Dulbecco's modified Eagle's medium (DMEM) containing 10% fetal bovine serum, 100 U/mL penicillin, and 100 μg/mL streptomycin, and then placed in a culture flask for 60 min to allow the heart fibroblasts to preferentially adhere to the bottom. The unattached and weakly attached cells, primarily cardiomyocytes, were collected and seeded onto plates. The cells were then incubated at 37°C and 5% CO_2_ in a humidified incubator. After 48 h, the cardiomyocytes that had adhered to the dish were used for subsequent experimental procedures. To establish a cardiomyocyte senescence model, the cells were treated with D‐gal (40 mg/mL) for 48 h.

### 4.5 Cell transfection

Primary mouse cardiomyocytes were transfected with miR‐203 mimic (miR10000236‐1‐5), miR‐203 inhibitor (miR20000236‐1‐5) and negative control (miR1N0000001–1‐5, miR2N0000001–1‐5) respectively (RiboBio). The transfection process involved the addition of reagents into Opti‐MEM (Gibco) and transfection into cardiomyocytes using X‐treme Reagent (Invitrogen). In another set of experiments, primary mouse cardiomyocytes were transfected with oe‐PARP1, oe‐NC (IBSBIO), or co‐transfected with oe‐PARP1 + mimic203 respectively. Transfection reagents and Lipofectamine 2000 (Invitrogen) were mixed separately with Opti‐MEM and subsequently applied to the cells. The cells were incubated at 37°C for 6 h, then treated with D‐gal (40 mg/mL) for 48 h.

### 4.6 Echocardiographic analysis

The mice were anesthetized with 0.2 g/kg of avertin (Sigma) through intraperitoneal injection and placed in the supine position on a temperature‐controlled ultrasonic table set at 37°C. Transthoracic echocardiography was performed using a Vevo®2100 High‐Resolution Imaging System (VisualSonics, FUJIFILM) ultrasound machine equipped with a 40.0‐MHz phase array transducer. M‐mode recordings were taken at the position of the papillary muscle. EF% and FS% were measured and calculated. Echo‐Doppler transmission flow imaging was performed in the apical 4‐chamber view by placing pulse‐wave Doppler at the mitral ring interval angle. The peak E velocity of the mitral ring was measured during early filling, while the peak A velocity was measured during the late diastolic period.

### 4.7 Histopathological and morphometric analysis

Histopathological changes and collagen distribution were evaluated by hematoxylin and eosin (HE) and Masson trichrome staining, respectively. Heart tissues were fixed with 4% paraformaldehyde, embedded in paraffin, and cut into 5 μm‐thick sections. Tissue sections were stained with HE reagent and Masson trichrome staining kit purchased from Solarbio Co. Images were captured using an optical microscope (Carl Zeiss Microscopy) and analyzed using Image J software.

### 4.8 SA‐β‐gal staining

SA‐β‐gal staining was performed using the senescence β‐galactosidase staining kit (Cell Signaling Technology) according to the manufacturer's instructions. Briefly, cells or frozen heart tissue sections were fixed and washed with phosphate‐buffered saline (PBS) twice. Then, 1 mL of β‐galactosidase staining solution was added to the cells or tissue sections, and they were incubated in a dry incubator (without CO_2_) at 37°C for 48 h. The stained samples were observed using a light microscope (Carl Zeiss Microscopy, Jena, Germany) and images were captured.

### 4.9 Immuno‐FISH

Tissue sections were fixed in 4% paraformaldehyde. After immunostaining, fluorescent in situ hybridization (FISH) of miR‐203 was performed using the RiboTM Fluorescent In Situ Hybridization Kit (RiboBio) according to the manufacturer's instructions. Briefly, samples were incubated in a hybridization buffer (37°C, 30 min) and then in a hybridization buffer containing a 10 μM miR‐203 FISH probe. After incubating at room temperature for 12 h, samples were washed in 0.1% Tween‐20 in 4 × SSC, 2 × SSC, and PBS. DAPI was used to label the cell nuclei. Finally, samples were imaged and collected using confocal laser microscopy.

### 4.10 Seahorse mitochondrial OCR analysis

The Seahorse XFe96 Analyzer (Agilent) was used to detect OCR. Primary mouse cardiomyocytes were seeded on a detection plate for 24 h and maintained until about 90% confluency. Cells were transfected with mimic‐203/inhibitor‐203 and incubated with 40 mg/mL D‐gal for 48 h. Cells were washed with Seahorse XF base medium and then incubated in 180 μL of medium per well (37°C, 1 h). The drug conditions were as follows: oligomycin (Port A; 1.5 μM), FCCP (Port B; 1 μM), and Rot/AA (Port A; 0.5 μM). Data were acquired after running the assay.

### 4.11 Mitochondrial membrane potential assay using JC‐1 staining

The JC‐1 staining assay kit (Beyotime) was used to measure mitochondrial membrane potential (∆Ψm). JC‐1 is a fluorescent probe widely used for detecting ∆Ψm. It emits red fluorescence at high ∆Ψm and green fluorescence at low ∆Ψm. In brief, primary cardiac cells were incubated with JC‐1 (37°C, 20 min), washed three times, and immediately observed and imaged using laser scanning confocal microscopy at emission wavelengths of 530 and 590 nm.

### 4.12 Reactive oxygen species (ROS) detection

The ROS assay kit (Solarbio) was used to detect ROS levels in primary cardiac cells cultured in a 24‐well plate. DCFH‐DA was diluted 1:1000 in serum‐free culture medium to a final concentration of 10 μM. After removing the culture medium, 250 μL of the diluted DCFH‐DA was added to each well and incubated at 37°C for 20 min. The cells were then washed three times with serum‐free culture medium and observed using a fluorescence microscope.

### 4.13 NAD^+^/NADH detection

The NAD^+^/NADH assay kit (WST‐8 method) from Beyotime is a colorimetric assay that utilizes the WST‐8 reagent to measure the amounts, ratios, and total levels of NAD^+^ and NADH in cells, tissues, or other samples. Briefly, 10–30 mg of cardiac tissue was homogenized using a homogenizer and then mixed with 400 μL of NAD^+^/NADH extraction buffer at room temperature. The mixture was then centrifuged at 12,000 g at 4°C for 5–10 min, and the supernatant was collected for the assay according to the manufacturer's instructions. The NAD^+^ and NADH concentrations as well as the NAD^+^/NADH ratio in the tissue samples were calculated based on a standard curve.

### 4.14 GEO dataset analysis

Gene expression data were obtained from the GEO database (GSE72890). The detection of differentially expressed genes (DEGs) was conducted using the “Deseq2” R package, with threshold values set at *p* < 0.01 and |log2 FC| > 1. DEGs were intersected with human aging‐related genes in the Aging Atlas (https://ngdc.cncb.ac.cn/aging/age_related_genes). The “clusterProfiler” R package was used to determine the underlying biological mechanisms of identified intersection genes. For Gene Ontology (GO) enrichment analysis, a threshold of *p*‐value <0.05 was applied to determine statistically significant enrichment. The “Goplot” R package was used to visualize genes linked to mitochondrial energy metabolism and NAD^+^ functions.

### 4.15 Luciferase reporter assay

Luciferase reporters containing wild‐type or mutated 3′UTR of PARP1 plasmid were constructed using psi‐CHECK2 vectors (Promega). Co‐transfection was performed using 0.5 μg of luciferase vector along with either miR‐203 mimic, miR‐203 inhibitor, or a negative control (scrambled sequence of miR‐203) for 5 h. After 48 h post‐transfection, cells were collected and lysed, followed by an assessment of luciferase activities using the Dual‐Luciferase Reporter Assay System (Promega). Renilla luciferase reporters served as an internal control.

### 4.16 RNA isolation and quantification

Total RNA was extracted from whole blood and cardiac tissue using TRIzol reagent (Invitrogen). The RNA samples were quantified using a NanoDrop ND‐8000 (Thermo Fisher Scientific). The RNA was then reverse transcribed into cDNA using a reverse transcription kit (Takara). Real‐time PCR was performed using SYBR Green (04913914001; Roche), with U6 used as an internal control for microRNA‐203 in cardiac tissue. The primer sequences are as follows:

MiR‐203 (Mouse).

Forward: 5′‐GGCGGTGAAATGTTTAGG‐3′.

Reverse: 5′‐CAGTGCGTGTCGTGGAGT‐3′.

U6 (Mouse).

Forward: 5′‐GCTTCGGCAGCACATATACTAAAAT‐3′.

Reverse: 5′‐CGCTTCACGAATTTGCGTGTCAT‐3′

### 4.17 Western blot analysis

The treated myocardial cells or myocardial tissue were lysed in RIPA buffer (Solarbio Co). The heart tissue was lysed in the total protein extraction kit (Wanleibio, China). Protein samples (60–100 μg/well) were separated by electrophoresis and transferred to nitrocellulose membranes (PALL). The membranes were blocked with 5% skim milk, and incubated with primary antibodies against p53 (Cat# 2524S, CST), p21 (Cat# ab188224, Abcam), and PARP1 (Cat# ab191217, Abcam) at 4°C for 12 h, with GAPDH as an internal control (Cat# TA‐08, ZSGB‐BIO). The membranes were then incubated with secondary antibodies at room temperature for 1 h. The target bands were analyzed using the Odyssey Infrared Imaging System (LI‐COR).

### 4.18 Quantification and statistical analysis

All statistical tests were analyzed using SPSS version 25.0 (IBM Corporation) or GraphPad Prism version 9.0 (GraphPad Software). Statistical analysis was performed using two‐way ANOVA or one‐way ANOVA followed by Tukey's post‐hoc test for multi‐group comparisons. Student's unpaired two‐tailed *t*‐test was used to compare the two groups. *p*‐value of <0.05 was considered statistically significant.

## AUTHOR CONTRIBUTIONS

Y.Z. and X.L. designed the studies, and L.Z. and Y.L. performed most of the experiments. P.T., M.D., L.J., H.L., H.S., and J.H. performed experiments on transgenic mice. H.X., H.S., R.X., Z.S., and H.L. assisted with cell culture, western blot, and PCR experiments. H.L. and Z.C. collected clinical samples. L.J. and H.L. generated transgenic mice. L.Z. and Y.L. analyzed the data and P.T. and P.K. wrote the manuscript.

## CONFLICT OF INTEREST STATEMENT

The authors declare no competing interests.

## Supporting information


Figure S1.

Figure S2.

Figure S3.

Figure S4.

Figure S5.

Table S1.

## Data Availability

The data that support the findings of this study are available from the corresponding author upon reasonable request.
