# Supplementary figures and images for: MiR‐203 improves cardiac dysfunction by targeting PARP1‐NAD + axis in aging murine
Source: Aging Cell. 2023 Dec 14;23(3):e14063. doi: 10.1111/acel.14063 (PMC10928583; doi:10.1111/acel.14063)

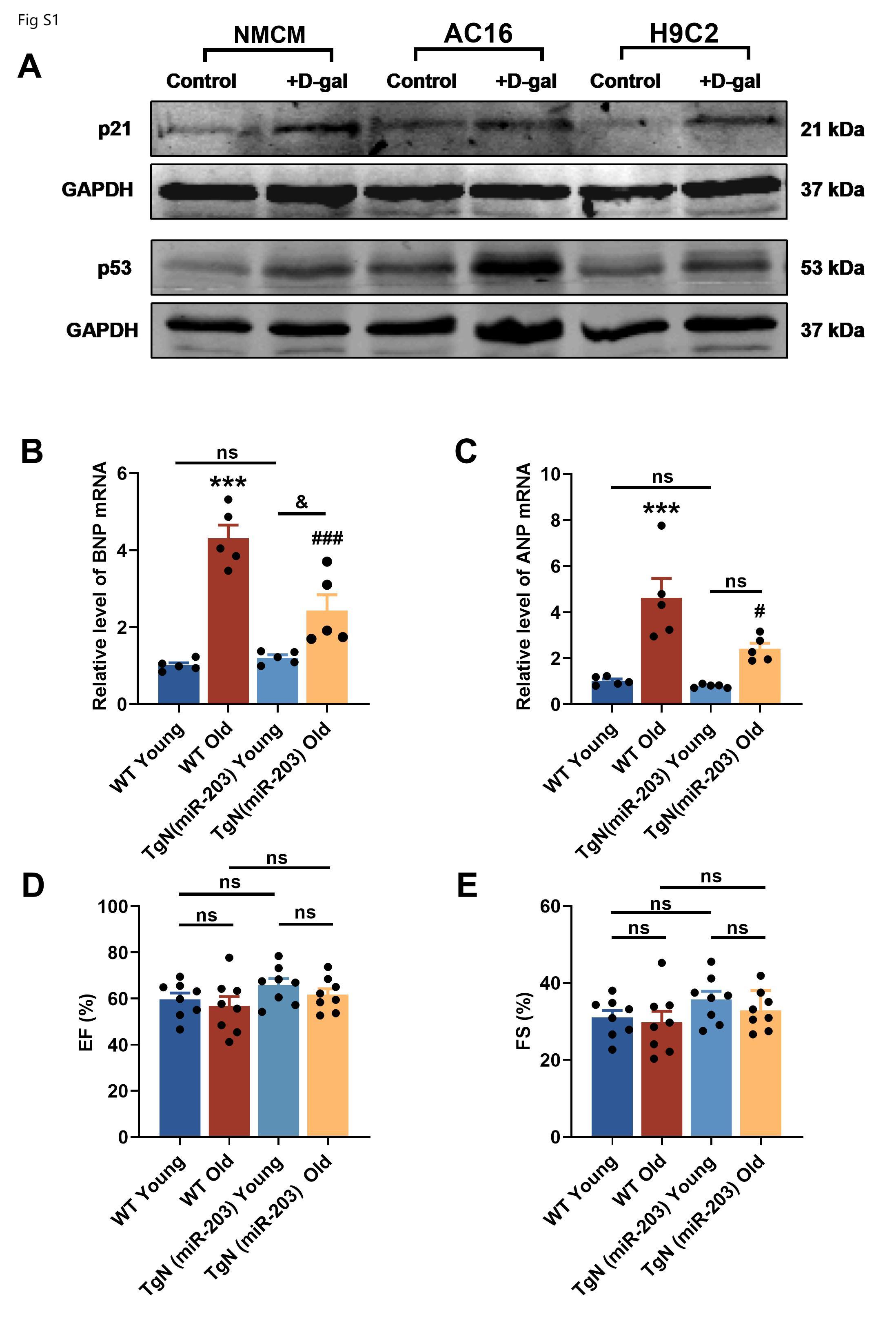

Supplement: Supplementary file 1 — Figure S1. [file ACEL-23-e14063-s006.jpg]

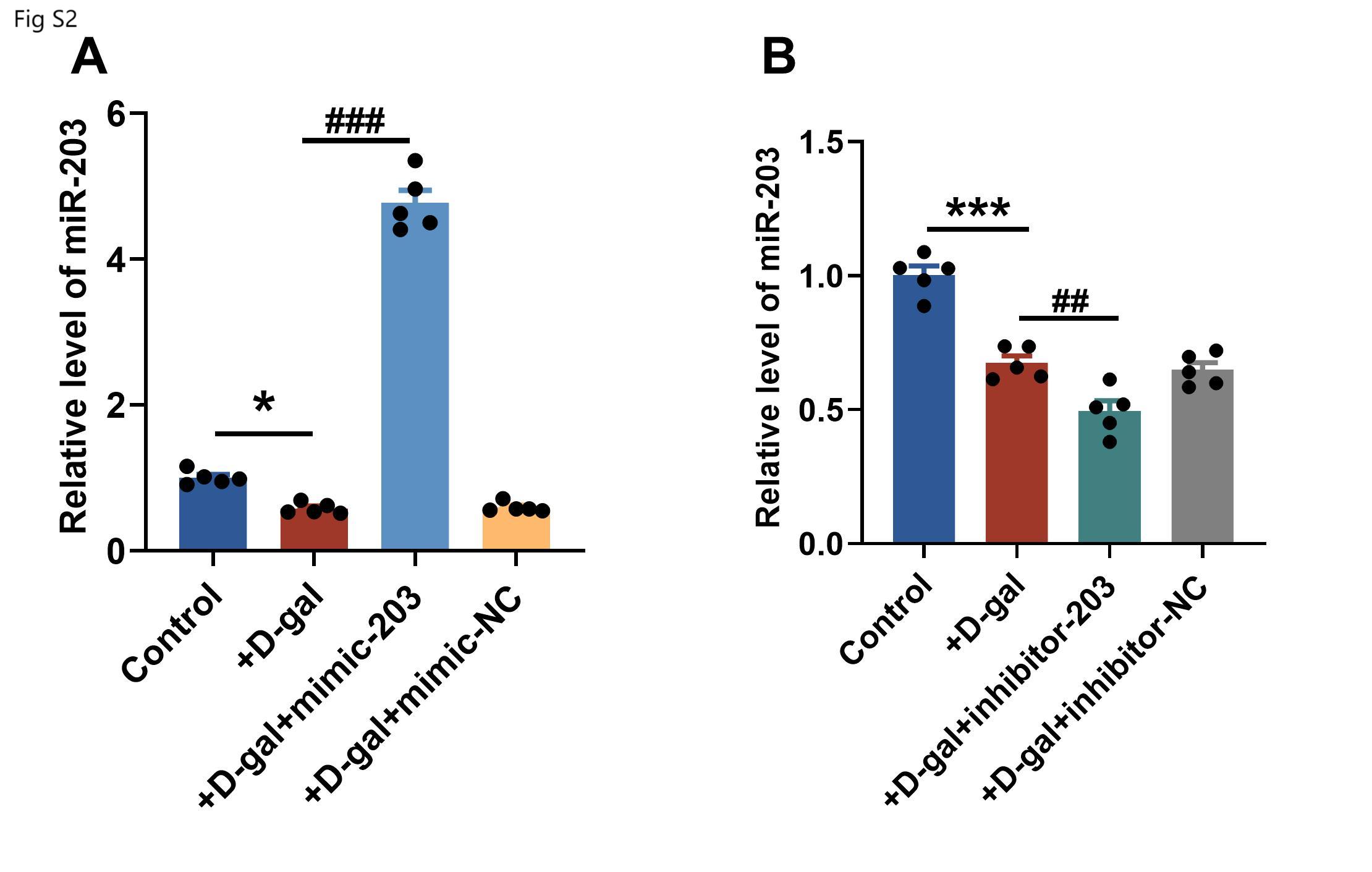

Supplement: Supplementary file 2 — Figure S2. [file ACEL-23-e14063-s001.jpg]

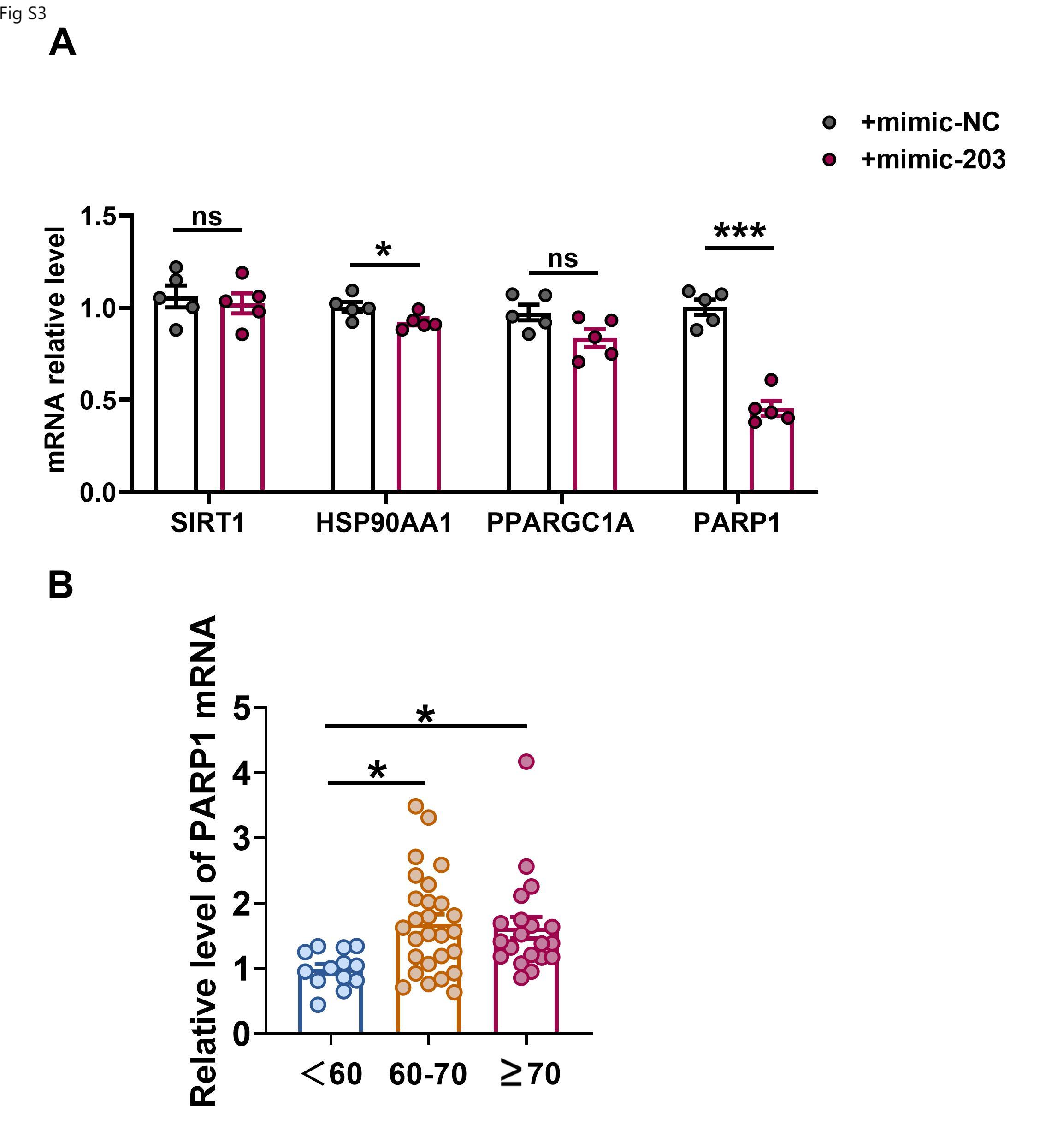

Supplement: Supplementary file 3 — Figure S3. [file ACEL-23-e14063-s004.jpg]

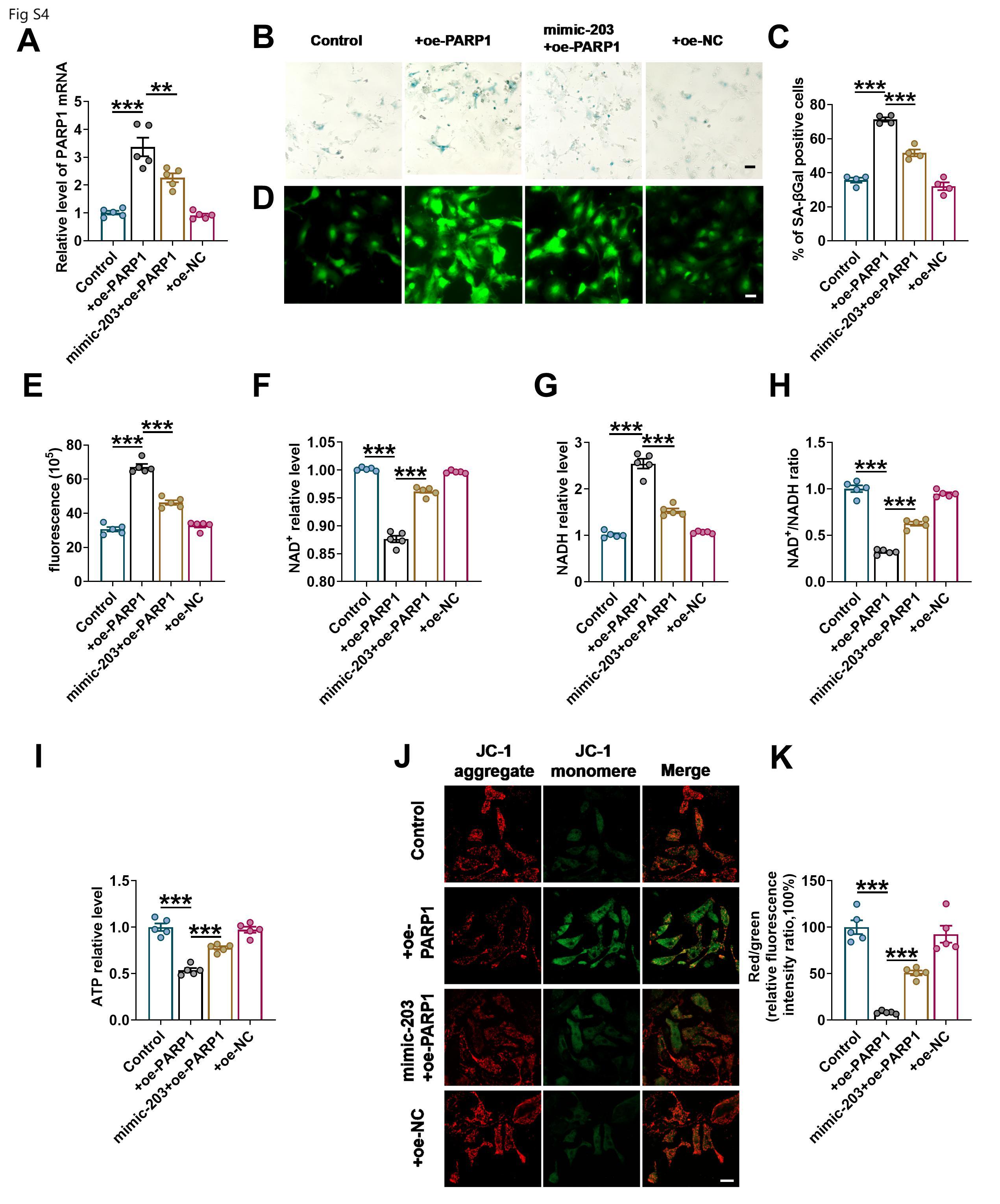

Supplement: Supplementary file 4 — Figure S4. [file ACEL-23-e14063-s005.jpg]

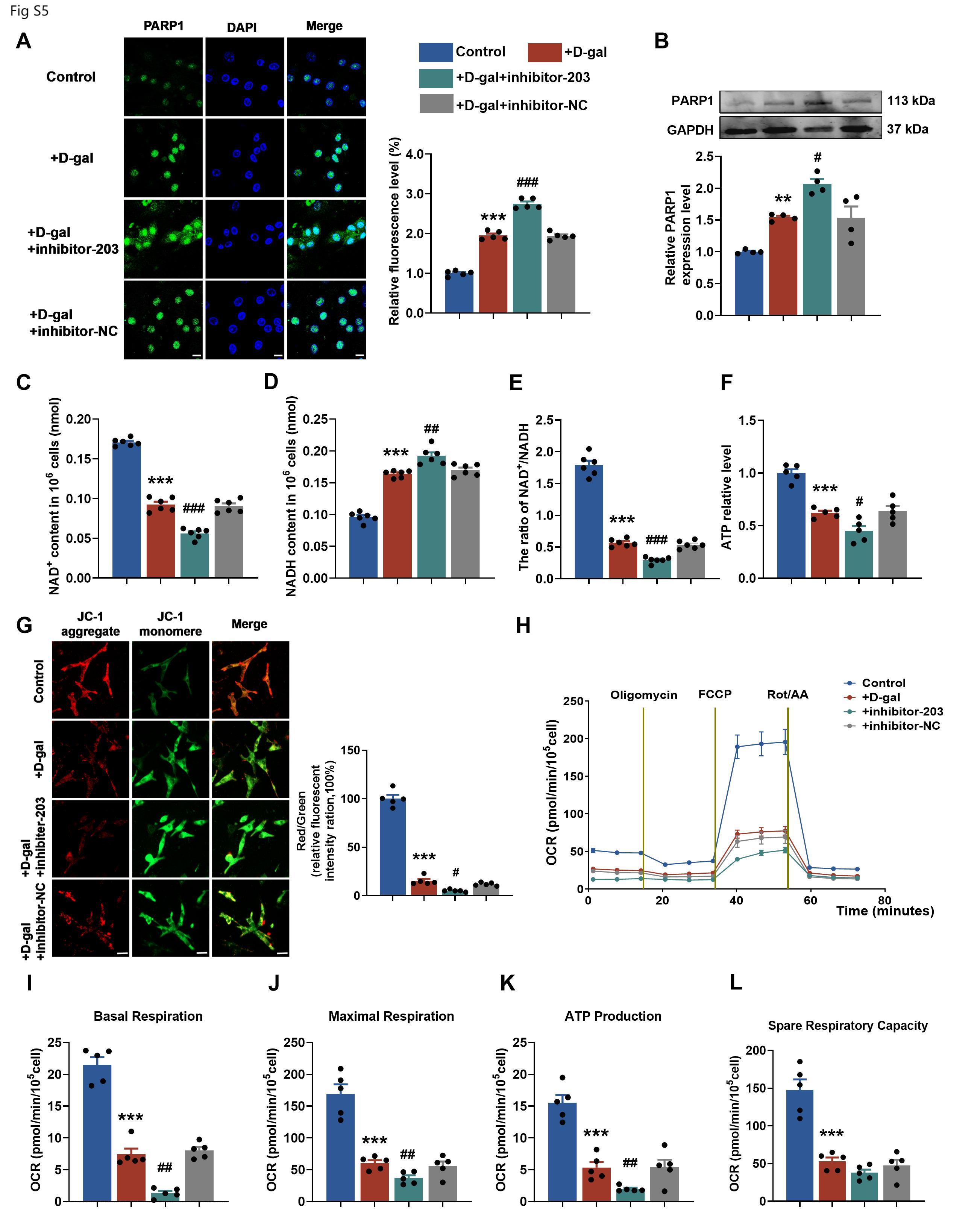

Supplement: Supplementary file 5 — Figure S5. [file ACEL-23-e14063-s003.jpg]
