# Supplementary material for: MiR‐203 improves cardiac dysfunction by targeting PARP1‐NAD + axis in aging murine
Source: Aging Cell. 2023 Dec 14;23(3):e14063. doi: 10.1111/acel.14063 (PMC10928583; doi:10.1111/acel.14063)
Supplement: Supplementary file 6 — Table S1. [file ACEL-23-e14063-s002.docx]

| **Supplemental table1. Mmu miR-203-3p binding target prediction** | | |
| --- | --- | --- |
| Gene | Binding site | Minimum Free Energy |
| PARP1 | 3 | -20.2 kcal/mol |
|  |  | -19.2 kcal/mol |
|  |  | -18.0 kcal/mol |
| Hsp90aa1 | 0 |  |
| Ppargc1a | 2 | -17.4 kcal/mol |
|  |  | -17.3 kcal/mol |
| Sirt1 | 4 | -15.2 kcal/mol |
|  |  | -14.6 kcal/mol |
|  |  | -14.4 kcal/mol |
|  |  | -13.4 kcal/mol |
| Dysregulation genes in aging mice associated with two biological processes of mitochondrial energy metabolism and NAD^+^ functions. | | |
